# Comparative Efficacy and Pharmacokinetic Parameters of Micronized Creatine Monohydrate (KleanCREATINE™) in Active Men: A Randomized, Controlled Clinical Study

**DOI:** 10.7759/cureus.103091

**Published:** 2026-02-06

**Authors:** Pravin Aggarwal, Ramshyam Agarwal

**Affiliations:** 1 Quality Assurance and Quality Control, Lasons India Pvt. Ltd., Mumbai, IND; 2 General Medicine, Lokmanya Medical Research Centre and Hospital, Pune, IND

**Keywords:** bioavailability, creatine monohydrate, endurance, muscle mass, strength

## Abstract

Introduction

This two-phase clinical study evaluated KleanCREATINE™ (micronized creatine monohydrate) for its pharmacokinetic properties, safety, and efficacy in active men.

Materials and methods

Phase I was a randomized, open-label, crossover study comparing its pharmacokinetic profile with a marketed creatine monohydrate in 10 healthy men. Phase II was a randomized, double-blind, parallel-arm trial in 52 active men over eight weeks, assessing the safety and efficacy of KleanCREATINE™ on muscle strength, endurance, hypertrophy, body composition, and perceived exertion.

Results

In Phase I, KleanCREATINE™ demonstrated better pharmacokinetics profile with higher C_max _(8.10 vs 4.80 nmol/UI), quicker T_max_ (2 vs 3 hours), and greater relative bioavailability (160.42% in sequence one and 175.45% in sequence two) over the marketed formulation. Phase II revealed significant effectiveness of KleanCREATINE™ over eight weeks: significant reduction in body fat percentage (14.45%), increase in skeletal muscle (10.46%), and enhanced muscle hypertrophy. KleanCREATINE™ showed greater chest press endurance (60.05% vs 49.29%). Perceived exertion decreased more with KleanCREATINE™ (26.4% vs 13.46%). Both formulations demonstrated safety and tolerability, with no adverse effects reported.

Conclusions

Micronized KleanCREATINE™ exhibited faster absorption, higher bioavailability, and greater efficacy in improving strength, endurance, and body composition compared to standard creatine monohydrate, with an excellent safety profile. These findings suggest KleanCREATINE™ as a promising alternative for athletes and fitness enthusiasts.

## Introduction

Ergogenic aids are frequently utilized to enhance performance or boost the adaptations resulting from exercise training. Creatine, naturally occurring in some foods and synthesized in the body, is primarily stored in skeletal muscles. Creatine monohydrate, in the form of supplements, has been extensively studied. It serves as a critical energy source during high-intensity, short-duration activities, such as weightlifting and sprinting, by replenishing adenosine triphosphate (ATP) stores, contributing to improvements in muscle strength, endurance, and volumization, as well as increased fat-free mass and muscle morphology [1]. Creatine, converted to phosphocreatine in muscles by creatine kinase, is crucial for intracellular ATP production. Creatine supplementation enhances ATP and energy production during heavy anaerobic exercise, potentially increasing muscle power, repetitions, and exercise volume. This, in turn, may contribute to improved muscle performance and hypertrophy over the training period [2]. As an endogenous compound, creatine belongs to the guanidine phosphagen family and is primarily produced by the liver, kidneys, and pancreas. It can also be obtained exogenously through meat and seafood or commercially produced creatine supplements. Skeletal muscles contain 95% of creatine, with the brain and testes containing approximately 5%. The average intramuscular creatine pool is 120 mmol/kg of dry muscle mass, with a maximum of 160 mmol/kg [3].

Approximately 1%-2% of intramuscular creatine is converted to creatinine, a metabolic byproduct excreted in urine. To maintain normal creatine levels, the body requires daily replenishment of about 1-3 g. Creatine supplementation is particularly beneficial for vegetarians with lower intramuscular creatine stores and athletes engaged in intense training, who may require higher amounts of creatine [4]. Creatine monohydrate is the most common form of creatine supplementation, known for its optimal effectiveness and bioavailability in elevating plasma creatine, enhancing tissue creatine, and improving performance. Evidence-based research has focused on assessing the impact of creatine monohydrate, combined with exercise, on indicators of muscle/lean mass, muscle performance, and recovery, predominantly in young, healthy male adults [5].

After a thorough review of the existing literature, the International Society of Sports Nutrition (ISSN) has identified creatine as the most effective nutritional supplement for enhancing high-intensity exercise capacity and promoting lean body mass during training among athletes. Similar conclusions have been reached by the American Dietetic Association, Dietitians of Canada, and the American College of Sports Medicine in their position statements [6].

In addition to its performance-related benefits, creatine supplementation has demonstrated promising health and therapeutic advantages, especially in the aging population. Notably, creatine supplementation has been associated with positive effects such as recovery from exercise, rehabilitation from immobilization, reducing cholesterol and triglycerides, and managing blood lipid levels, mitigating fat accumulation in the liver, lowering the risk of cardiovascular disease, serving as an antioxidant, improving glycemic control, impeding the progression of certain forms of cancer and neurodegenerative diseases, enhancing strength and muscle mass, minimizing bone loss in some studies, improving functional capacity in osteoarthritic and fibromyalgia patients, enhancing cognitive function, particularly in older populations, and, in some instances, enhancing the effectiveness of certain anti-depressant medications [6,7].

In light of the compelling evidence supporting the multifaceted benefits of creatine supplementation on athletic performance, muscle health, and potential therapeutic advantages, our present study aims to contribute valuable insights into the pharmacokinetics, bioavailability, safety, and efficacy of creatine monohydrate supplementation.

## Materials and methods

Study design

This study was conducted in two phases with active male participants. Phase I was a randomized, open-label, crossover, controlled trial evaluating the single-dose pharmacokinetics, bioavailability, and safety of a creatine monohydrate supplement. Ten participants per group were enrolled after crossover, with detailed assessments following a single-dose administration. The study aimed to characterize the absorption, distribution, metabolism, and elimination of the supplement and monitor safety.

Phase II was a randomized, double-blind, parallel-arm, controlled trial assessing the safety and efficacy of supplementation on strength, power, muscle volumization, energy, and lean body mass over eight weeks. Fifty-two participants were equally divided into two groups and monitored. Efficacy outcomes were compared between groups. The study received ethical approval from the Ethics Committee of Lokmanya Medical Research Centre, Pune (ECR/175/Inst/MH/2013/RR-19), adhering to the Declaration of Helsinki and Good Clinical Practice guidelines. This clinical trial was registered with the Clinical Trial Registry of India (CTRI/2024/02/063205). Data collection occurred between March and June 2024 (Phase II).

Randomization of allocation of participants to groups in Phase II was performed using a computer-generated random sequence in a 1:1 allocation ratio generated by a qualified statistician. Allocation concealment was ensured using sequential numbers. The participants and investigators were blinded to group allocation. Both investigational products were identical in appearance.

Study participants

Inclusion Criteria

The study was exploratory and pragmatic in nature. Based on research judgement, the sample size for Phase I was 20 participants and 50 participants for Phase II. Male participants aged 18-40 years with BMI <30 kg/m² and in normal health were enrolled. Eligibility required regular resistance training for one or more year, no creatine supplementation in the last month, and availability for the entire study duration. Participants had to be able to fast for 14 hours, consume standard meals, and provide informed consent. Phase I participants could continue to Phase II after a seven-day washout period. All participants agreed for the follow-up as required by the study protocol.

Exclusion Criteria

Participants were excluded if they had contraindications to creatine supplementation, existing medical conditions (cardiac, respiratory, metabolic, etc.), or were using performance-enhancing drugs or medications affecting muscle biology. Those with renal or liver dysfunction, planning extended travel without fitness facility access, or any condition potentially confounding study outcomes were also excluded. The investigator's discretion was used to determine if any other factors might preclude a participant's ability to complete the study.

Objectives

Phase I

*Primary objective*: To evaluate and compare the pharmacokinetics and relative bioavailability of micronized creatine monohydrate (KleanCREATINE™) versus a marketed creatine monohydrate formulation.

*Secondary objective*: To assess the safety and tolerability of the investigational products following single-dose administration.

Phase II

*Primary objective*: To evaluate and compare the effects on muscle strength and volumization in healthy male participants of micronized creatine monohydrate (KleanCREATINE™) versus a marketed creatine monohydrate formulation.

*Secondary objective*: Secondary objectives included evaluating changes in muscle endurance, strength, power, volume, energy, lean body mass, body composition, and anthropometric parameters. Additionally, the study assessed the safety and tolerability of the investigational products by monitoring adverse events throughout the trial.

Clinical study procedure

This randomized, controlled clinical trial evaluated the pharmacokinetics, bioavailability, safety, and efficacy of creatine monohydrate supplements in enhancing strength, power, muscle volumization, energy, and lean body mass in active males. The study was conducted in two phases. Participants had engaged in regular resistance training for at least one year and continued their habitual training throughout the study. Participants were instructed to maintain their usual diet and supplementation practices under free-living conditions.

**Figure 1:**
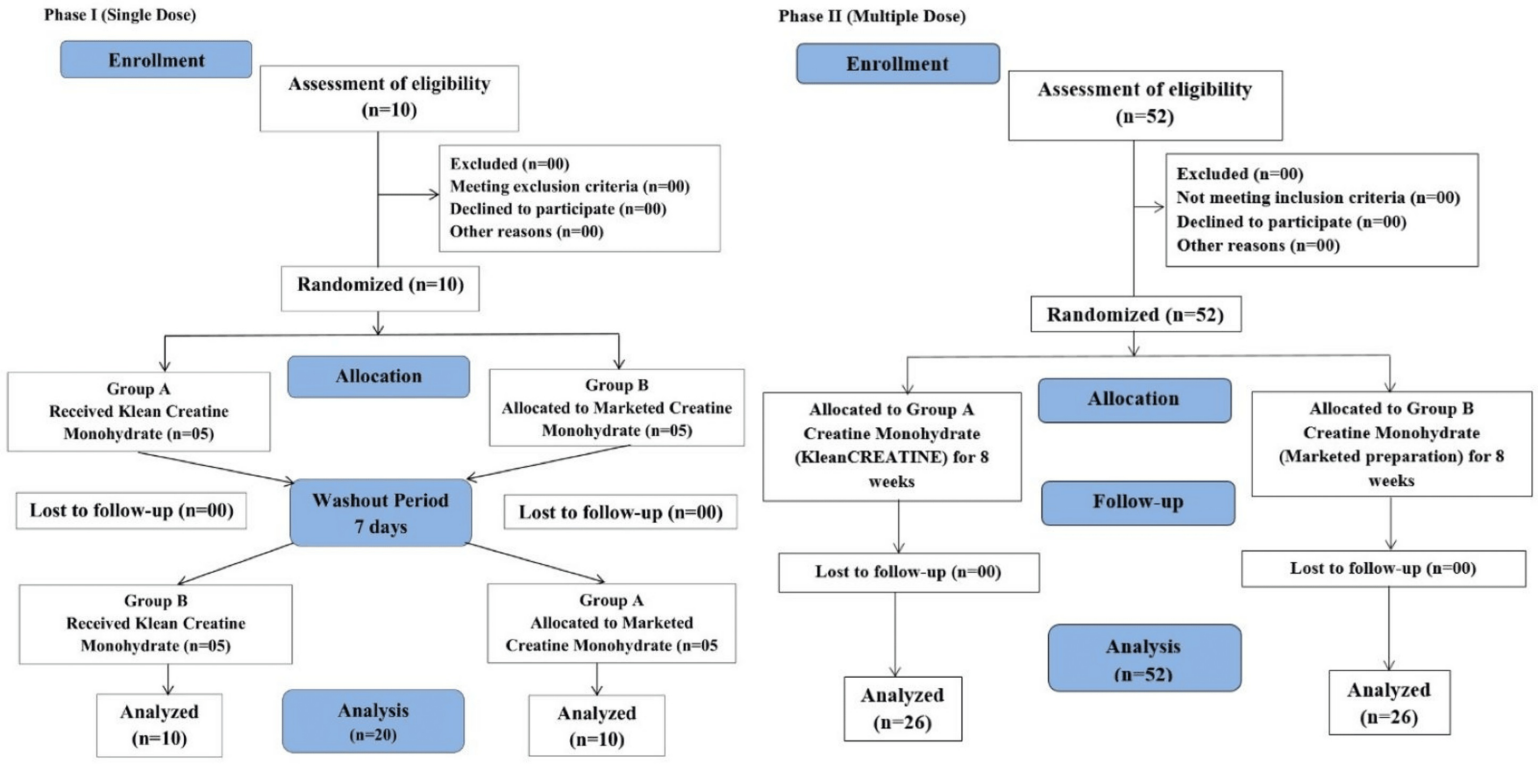
CONSORT diagram for Phase I and Phase II of the study CONSORT=Consolidated Standards of Reporting Trials

In Phase I (single-dose pharmacokinetics), 10 participants were enrolled per arm, divided into two groups of five each (Figure 1). Group A received KleanCREATINE™, while Group B received a marketed preparation. After a seven-day washout period, the groups crossed over. Participants fasted overnight, with breakfast provided three hours before administration. Each participant consumed 3 g of the assigned product in 240 ml water. Meals were provided every two hours post-administration. Blood samples were collected pre-dose and at 0.5, 1, 2, 3, 4, 5, and 6 hours post-dose. These samples were analyzed for creatine monohydrate using enzyme-linked immunosorbent assay (ELISA). Pharmacokinetic parameters calculated included F, C_max_, T_max_, AUC_0-t_, AUC0_-∞_, t_1/2_, and K_el_.

Phase II (multiple dose) involved 52 participants (26 per arm) over eight weeks (Figure 1). Participants consumed a daily dose of 3 g of the assigned product in 240 ml water/fruit juice before a meal and were advised to limit caffeine intake. Assessments included muscle strength (leg press and chest press 1RM), muscle endurance (reps to fatigue at 50% 1RM), hypertrophy (measurements of biceps, triceps, quads, and hamstrings), perceived exertion (scale of 1-10), and body composition and anthropometric parameters. Strength, endurance, hypertrophy, and perceived exertion were assessed on day one, weeks four and eight, while body composition and anthropometrics were measured on day one and week eight. Safety monitoring included clinical and physical examinations at screening, baseline, week four, and week eight, with adverse events (AEs) and serious adverse events (SAEs) recorded throughout.

Blood samples were collected via intravenous catheter or direct venipuncture, centrifuged, and plasma separated and stored at -30°F until analysis. Pharmacokinetic data were processed using Phoenix® WinNonlin® software, correcting post-dose concentrations by subtracting baseline levels. This design allowed a comprehensive evaluation of immediate pharmacokinetics and longer-term efficacy and safety of creatine monohydrate supplementation in active males, with Phase I's crossover design controlling individual variability, and Phase II detailing supplement effects across eight weeks.

Statistical analysis

The study was exploratory and pragmatic in nature. Based on research judgement, a sample size of 20 participants in Phase I and 50 participants in Phase II was selected. Statistical analyses were conducted to compare between-group differences and within-group changes across predefined efficacy and safety endpoints. 

Phase I: Noncompartmental pharmacokinetic analysis was performed using Phoenix® WinNonlin® software (Certara, Inc., Princeton, NJ, USA). Key parameters (C_max_, T_max_, K_el_, t_1/2_, area under the curve (AUC)) were calculated for each group. Descriptive statistics (mean±SD) were used for demographic data.

Phase II: Normality was assessed using Kolmogorov-Smirnov test. Between-group comparisons utilized independent t-tests for anthropometric parameters, body composition, and muscle strength changes. Perceived exertion was evaluated using Mann-Whitney (between-group) and Wilcoxon tests (within-group). All statistical analyses were conducted using SPSS software Version 30.0 (IBM Corp, Armonk, NY).

## Results

Phase I (single-dose pharmacokinetics and bioavailability)

Assessment of Demographic Characteristics and Lifestyle Habits

Ten male participants (average age: 29.3 years, average BMI: 25.84 kg/m^2^) completed the study. Among the participants, two reported alcohol consumption, one smoked, and six consumed caffeine daily, all of whom refrained from these habits during the study. Additionally, nine followed a nonvegetarian diet, while one adhered to a vegetarian diet. Participants had 1.7±0.63 years of resistance training experience (Table 1).

**Table 1:** Baseline demographic and clinical characteristics of participants Age is represented as mean±SD.

Parameters	Observation
Phase I (n=10)	
Average Age (Years)	29.3±6.60
Gender	Male
Phase II (n=52)	
Average Age (Years)	27.37±5.40
Gender	Male

Assessment of Pharmacokinetic Parameters

**Figure 2:**
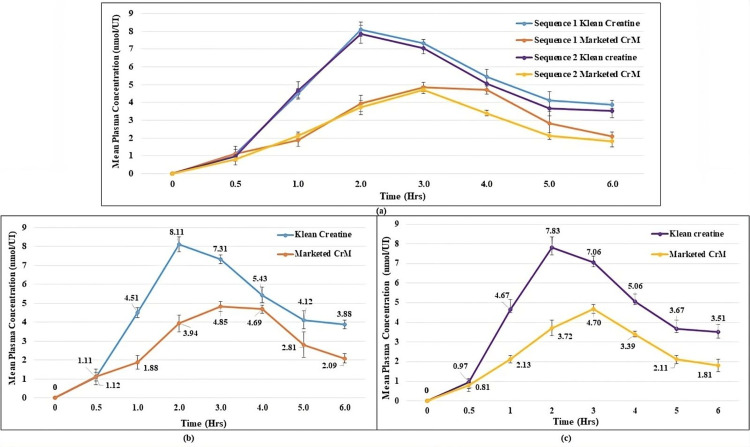
Mean plasma concentration vs. time curves for four randomized formulations of creatine monohydrate quantified: (a) overlay; (b) individual formulations (sequence 1) after oral administration of creatine monohydrate at the dose of 3 g in healthy male volunteers (mean±SD, n=5 healthy participants in each formulation group); (c) individual formulation (sequence 2) after oral administration of creatine monohydrate at the dose of 3 g in healthy male volunteers after crossover (mean±SD, n=5 healthy participants in each formulation group).

In the phase I crossover study, pharmacokinetic and bioavailability parameters of creatine monohydrate were evaluated in 10 healthy male participants. Group A (five participants) received KleanCREATINE™, while Group B (five participants) received a marketed creatine monohydrate (CrM) preparation. After a seven-day washout period, the groups switched interventions. The average plasma concentration (ng/ml) vs. time (hours) curve of creatine monohydrate is shown in Figure 2. Major pharmacokinetic parameters for both sequences are summarized in Table 2.

**Table 2:** Pharmacokinetic parameters for creatine monohydrate in the healthy male participants upon single-dose (3 g) oral administration of two formulations in a cross over study groups Sequence 1_ Marketed CrM is considered std reference for calculation of Relative F %. Sequence 2_ Marketed CrM is considered std reference for calculation of Relative F %. R^2^ Values are good in both the groups > 0.8. Data represented as Mean, (n=5 healthy participants in each group); C_max_±SD: Maximum observed plasma concentration± standard deviation; T_max_: Time to reach maximum observed plasma concentration; AUC_0-t_: Area under the plasma concentration-time curve from time zero to the last measurable concentration; AUC_0-∞_ : Area under the plasma concentration-time curve from time zero to infinity; % AUC_Extra_: Percentage of AUC extrapolated from the last measurable concentration to infinity; K_el_: Apparent terminal elimination rate constant; t_1/2_: Apparent terminal elimination half-life; Relative F: Relative bioavailability compared to a reference formulation.

PK Parameters	KleanCREATINE^™^	Marketed CrM
Sequence 1 (Mean)		
C_max_ (nmol/Ul)	8.10	4.80
T_max_ (hr)	2.00	3.00
AUC_0h-6h_ (hr*nmol/Ul)	30.80	19.20
AUC_0h-∞_ (hr*nmol/Ul)	50.50	24.80
% AUC Extra	39.10	22.60
K_el _(1/h)	0.20	0.40
t_1/2_ (h)	3.50	1.90
Relative F (%)	160.42	-
Sequence 2 (Mean)		
C_max_ (nmol/Ul)	7.80	4.70
T_max_ (hr)	2.00	3.00
AUC_0h-6h_ (hr*nmol/Ul)	29.30	16.70
AUC_0h-∞_ (hr*nmol/Ul)	45.70	23.30
% AUC Extra	36.00	28.20
K_el _(1/h)	0.20	0.30
t_1/2_ (h)	3.30	2.50
Relative F (%)	175.45	-

Sequence 1:The mean peak plasma concentration (C_max_) was highest in the KleanCREATINE™ group at 8.10 nmol/UI, compared to 4.80 nmol/UI for the marketed CrM, with T_max_ of 2 and 3 hours, respectively. AUC_(0h-6h)_ was 30.80 h* nmol/UI for KleanCREATINE™ and 19.20 h* nmol/UI for Marketed CrM, with %AUC_Extra_ of 39.10% and 22.60%. AUC_(0h-∞)_ was 50.50 h* nmol/UI and 24.80 hrnmol/UI, respectively. The half-life for KleanCREATINE™ and marketed CrM was 3.50 and 1.90 hours, with K_el_ values of 0.20 1/h and 0.40 1/h. relative The bioavailability of KleanCREATINE™ was 160.42%. Overall, KleanCREATINE™ demonstrated higher plasma concentration, AUC, and C_max_ compared to marketed CrM.

Sequence 2: The KleanCREATINE™ group showed the highest mean peak plasma concentration (C_max_) at 7.80 nmol/UI, compared to 4.70 nmol/UI for the marketed CrM, with T_max_ of two and three hours, respectively. AUC(0h-6h) was 29.30 h* nmol/UI for KleanCREATINETM and 16.70 h* nmol/UI for marketed CrM. AUC_(0h-∞) _was 45.70 h* nmol/UI and 23.30 hr* nmol/UI, respectively. Half-life was 3.30 hours for KleanCREATINE™ and 2.50 hours for marketed CrM, with K_el _values of 0.20 1/h and 0.30 1/h. KleanCREATINE™ relative bioavailability was 175.45%. Overall, KleanCREATINE™ demonstrated higher plasma concentration, AUC, and C_max_ compared to Marketed CrM.

Safety Assessment (Phase I)

During screening, all participants' hematological, liver function tests, and kidney function tests parameters were found to be within normal physiological ranges. No clinically significant changes were observed in blood pressure, pulse rate, respiratory rate, and oral temperature across all four groups throughout the study. All vital signs remained within normal limits in all participants in both the sequence of the study. All participants were compliant and showed excellent tolerability to the investigational products in both the sequence.

Phase II (multiple dose)

Assessment of Demographic Characteristics of the Phase II Participants

Fifty-two male participants (26 in each arm) completed the study. The average age was 27.37 years, comparable between groups. Among 52 participants, 28 reported alcohol consumption, 15 reported smoking, and 24 reported daily caffeine consumption, all of whom refrained during the study. Thirty-seven consumed nonvegetarian food and 15 had vegetarian food. Participants had 2.22±0.83 years of resistance training experience (Table 1).

Assessment of Anthropometric and Body Composition Parameters of the Phase II Participants

**Figure 3:**
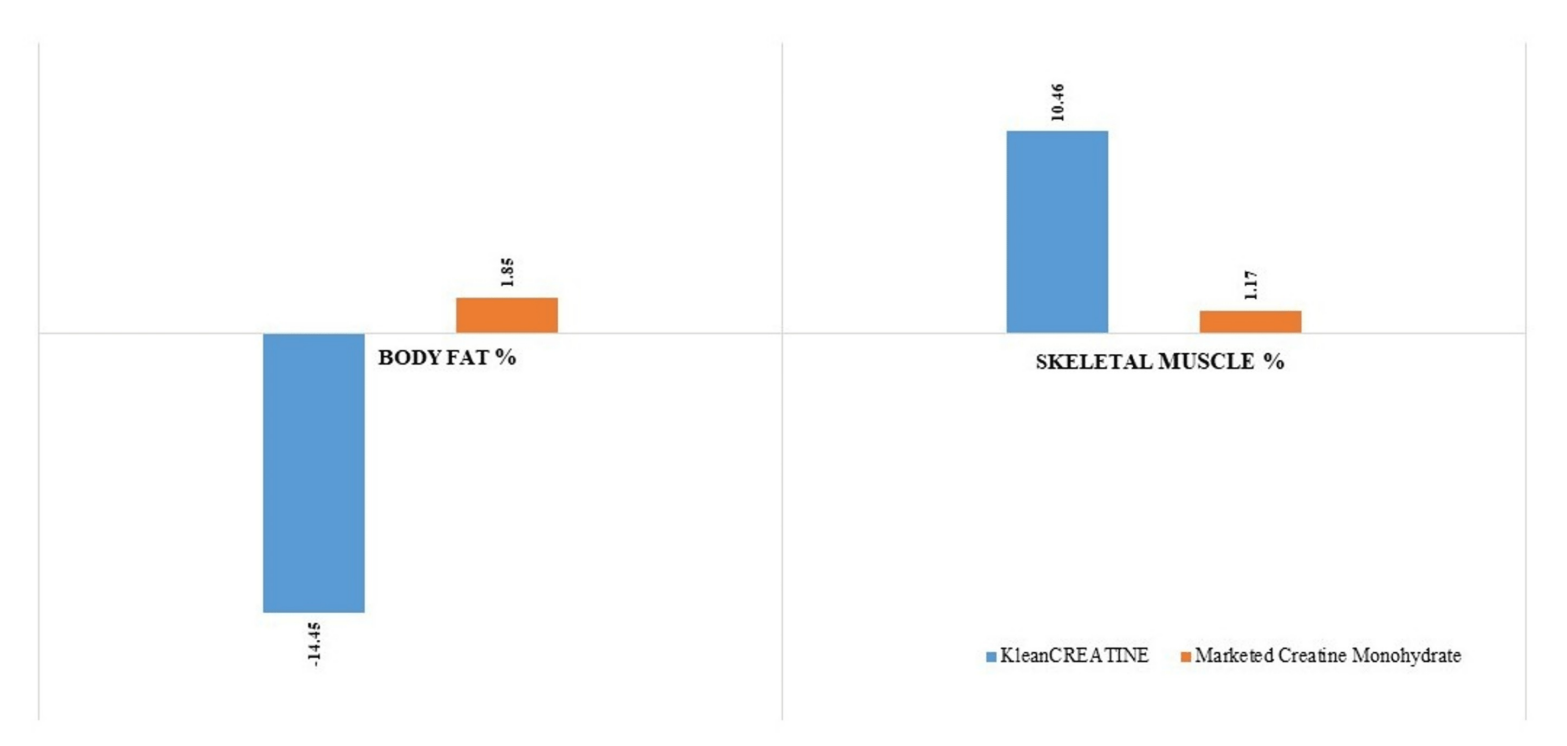
Assessment of changes in body composition through BIA between groups of the phase II participants BIA: Bioelectrical impedance analysis.

No significant changes in the body weight and BMI was observed in participants after eight weeks (Table 2). At screening, % body fat and % skeletal muscle was comparable. The KleanCREATINE™ group exhibited a statistically significant 14.45% reduction in body fat, while the marketed CrM group had a slight, non-significant 1.85% increase. The between-group comparison at week eight was significant. Regarding skeletal muscle %, the KleanCREATINE™ group showed a substantial 10.46% increase, which was statistically significant. In contrast, the marketed CrM group had a minor 1.17% increase, which was not statistically significant. These results indicate that the KleanCREATINE™ supplement was more effective than the marketed CrM in reducing body fat and increasing skeletal muscle mass over the eight-week study period (Figure 3 and Table 3).

**Table 3:** Assessment of changes in body composition through BIA between groups of the phase II participants Data is represented as mean±SD. Analysis was done using independent student t test (between group). Significant at p<0.05. BMI: Body mass index; SD: standard deviation; BIA: Bioelectrical impedance analysis

Assessment of anthropometric and body composition Parameters				
Duration	KleanCREATINE^™^ (n=26)	Marketed CrM (n=26)	P value	t-value
Weight (kg)				
Day 1	72.23±10.73	74.19±9.48	0.488	-0.698
Week 8	72.85±8.97	74.14±9.14	0.184	-1.348
P value	0.207	0.715	-	-
BMI (kg/m^2^)				
Day 1	24.66±3.64	25.41±3.64	-	-
Week 8	24.88±3.07	25.40±3.58	-	-
% Body fat				
Day 1	23.66±5.76	23.87±6.64	0.906	-0.118
Week 8	20.24±4.46 (14.45%)	24.31±6.36 (1.85%)	<0.001	4.815
% Skeletal muscle				
Day 1	45.77±3.67	45.75±4.78	0.987	0.016
Week 8	50.55±3.45 (10.46%)	46.28±4.11 (1.17%)	<0.001	-3.643

Assessment of Changes in Muscle Hypertrophy

**Figure 4:**
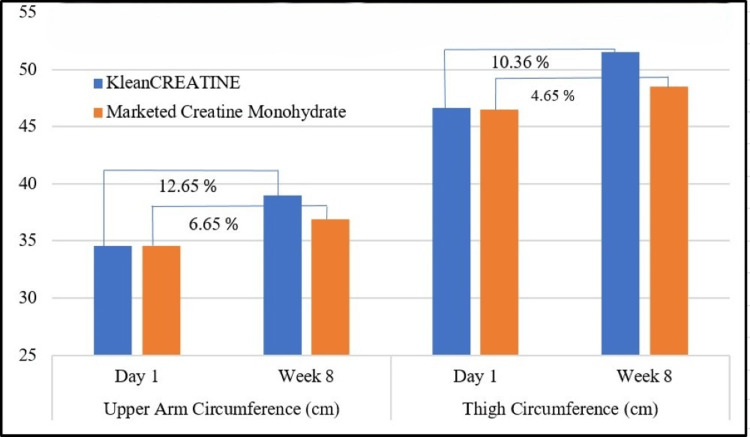
Assessment of changes muscle hypertrophy between groups of the phase II participants

For arm circumference, both groups started similarly but diverged by week eight, with KleanCREATINE™ group's arm circumference increasing by 12.65%, significantly higher than the 6.65% increase in the marketed CrM group. A similar trend was observed for thigh circumference, with the KleanCREATINE™ group increasing by 10.36% compared to 4.65% in the marketed CrM group. Overall, the KleanCREATINE™ group demonstrated more consistent and substantial increases in both arm and thigh circumference compared to the marketed CrM group over the eight-week study (Figure 4).

Assessment of Changes Muscle Strength

1RM (one-repetition maximum) measures the maximum weight lifted for one repetition. The chest press targets pectoral muscles, while the leg press focuses on quadriceps, hamstrings, and glutes. Phase II results showed differences between KleanCREATINE™ and marketed CrM groups. The KleanCREATINE™ group also demonstrated significant within-group improvements at both weeks four and eight, while the marketed CrM group only showed a significant increase at week eight.

**Figure 5:**
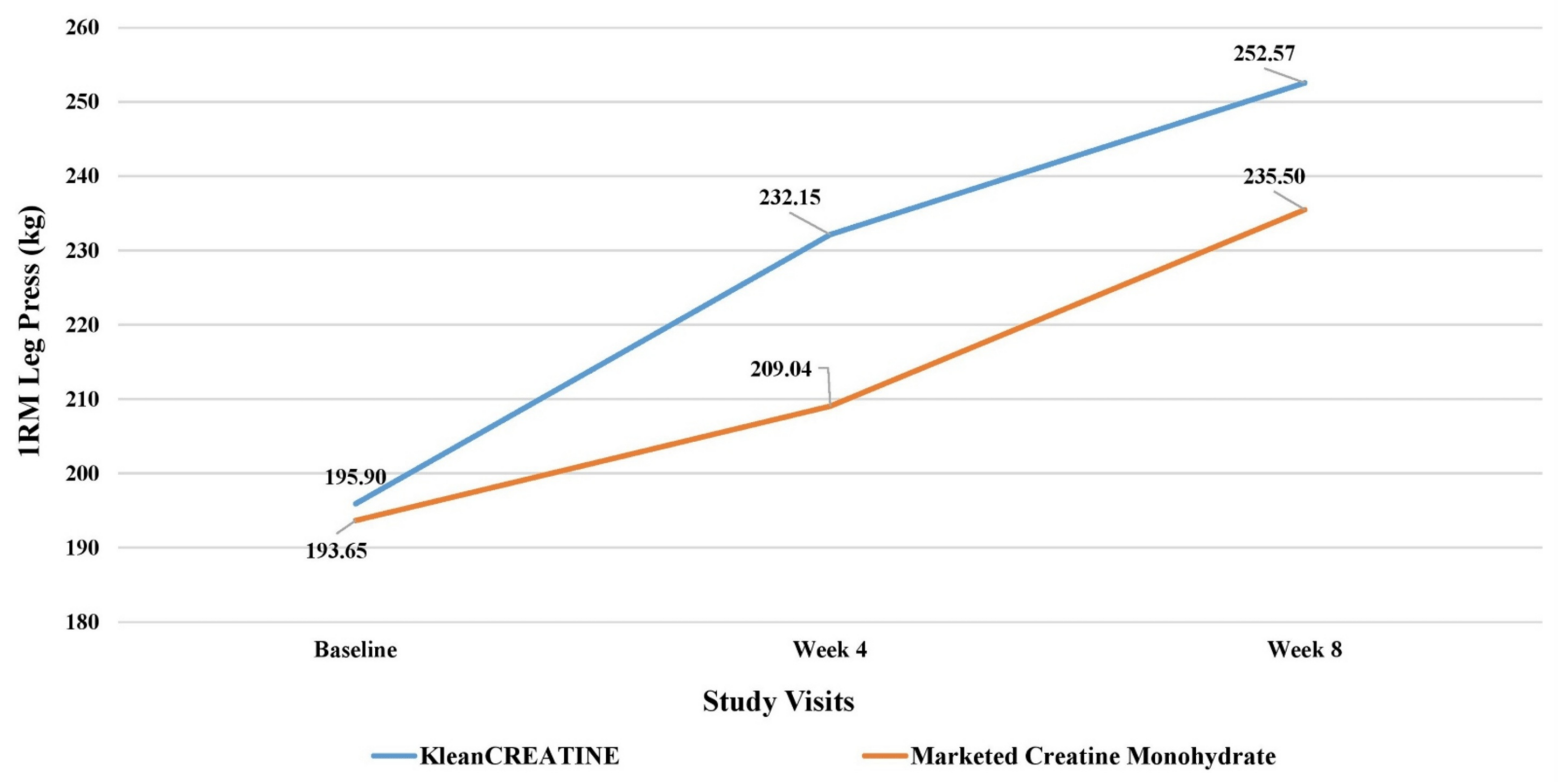
Assessment of muscle strength 1RM leg press (kg) of the phase II participants 1RM: One-repetition maximum.

**Figure 6:**
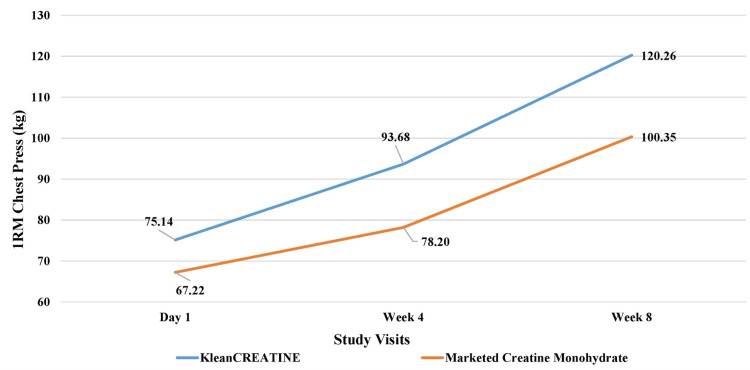
Assessment of muscle strength 1RM chest press (kg) of the phase II participants 1RM: One-repetition maximum.

For the 1RM chest press, the KleanCREATINE™ group had a significantly greater increase of 25% by week four, compared to 16% for the marketed CrM group. By week 8, the KleanCREATINE™ group reached a 60% increase, which was significantly higher than the 49% increase in the marketed CrM group. For the 1RM leg press, the KleanCREATINE™ group showed a 19% increase by week four, while the marketed CrM group only had an 8% increase, though the difference was not statistically significant. By week eight, the KleanCREATINE™ group further increased to 29%, which was higher than the 22% increase in the marketed CrM group. Both groups showed significant within-group improvements at weeks four and eight (Figures 5, 6).

Assessment of Muscle Endurance (Reps to Fatigue at 50% 1-RM)

Total reps to fatigue sums all repetitions performed across leg press and bench press exercises to gauge the overall muscle endurance. For leg press reps to fatigue, both groups improved significantly by week four and eight compared to day one, with no between-group significant differences at week four and eight. For chest press reps to fatigue, the KleanCREATINE™ group showed significant improvements from day one to weeks four and eight, while the marketed CrM group also improved significantly. Overall, the KleanCREATINE™ group exhibited a greater magnitude of improvement compared to the placebo group at both weeks four and eight.

**Figure 7:**
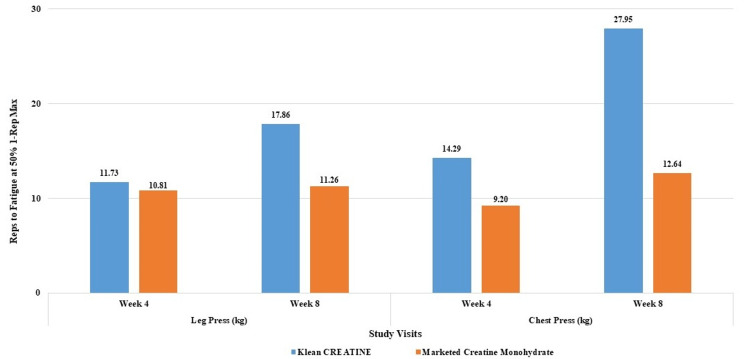
Assessment of percent improvement in leg press and chest press reps to fatigue at 50% 1RM between groups 1RM: One-repetition maximum.

In terms of total reps to fatigue, the groups showed significant between-group differences on day one, but both improved significantly by weeks four and eight, with the KleanCREATINE™ group performing significantly better at week eight. Overall, the KleanCREATINE™ group demonstrated more consistent and significant increases in muscle endurance compared to the marketed CrM group (Table 4 and Figure 7).

**Table 4:** Assessment of changes in reps to fatigue at 50% of 1RM between groups of the phase II participants Data is represented as mean±SD. Analysis was done using independent student t test (between group). Significant at p<0.05. SD: standard deviation.

Parameter	Day 1			Week 4		Week 8		
	Klean CREATINE^™^ (n=26)	Marketed CrM (n=26)		Klean CREATINE^™^ (n=26)	Marketed CrM (n=26)	Klean CREATINE^™^ (n=26)		Marketed CrM (n=26)
Reps to Fatigue at 50% 1-Rep Max (Number of repetitions)								
Leg Press Reps to Fatigue at 50% 1-Rep Max	3.77±0.57	4.27±0.62		4.21±0.57	4.73±0.55	4.44±0.48		4.75±0.53
% Change	-	-		11.73%	10.81%	17.86%		11.26%
P value	0.004			0.738		0.055		
t-value	-3.027			0.336		-1.967		
Chest Press (kg) Reps to Fatigue at 50% 1-Rep Max	3.10±0.40	3.35±0.52		3.54±0.40	3.65±0.51	3.96±0.37		3.77±0.92
% Change	-	-		14.29%	9.20%	27.95%		12.64%
P value	0.059			0.299		0.063		
t-value	-1.931			-1.049		-1.903		
Total reps to fatigue (sum of all Reps to Fatigue)								
Total reps to fatigue (sum of all Reps to Fatigue)	6.87±0.63		7.62±0.86	7.75±0.68	8.38±0.83	8.40±0.58	8.13±0.59	
% Change	-		-	12.89%	10.10%	22.41%	6.82%	
P value	0.001			0.432		<0.001		
t-value	-3.586			-0.793		-5.126		

Assessment of Rating of Perceived Exertion

**Figure 8:**
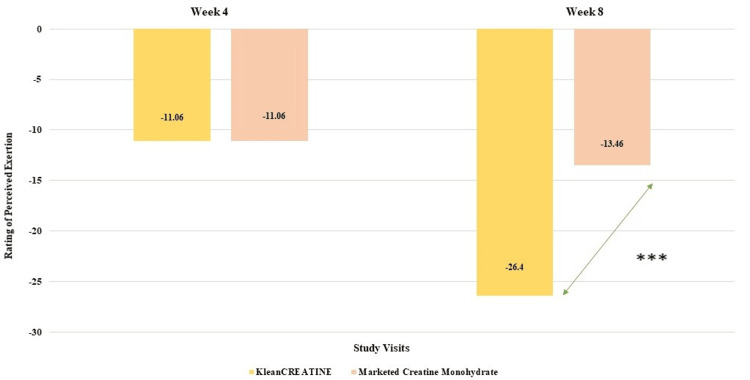
Assessment of changes in perceived exertion

The rating of perceived exertion (RPE) measures how hard a workout feels to participants on a scale of 1 to 10, with higher scores indicating more difficult workouts. On day one, both groups reported identical RPE. By week four, both experienced an 11.06% decrease in perceived exertion, with no significant difference between groups. At week eight, KleanCREATINE™ group showed a 26.4% reduction in RPE, while marketed CrM reported a 13.46% reduction. The difference between groups at week eight was statistically significant. Within-group analyses showed significant reductions for both groups at Weeks four and eight. Overall, KleanCREATINE™ demonstrated a more substantial decrease in perceived exertion compared to the marketed CrM throughout the study (Table 5 and Figure 8).

**Table 5:** Assessment of changes in perceived exertion after workout between groups of the phase II participants Data is represented as mean±SD. Analysis was done using Mann-Whitney test (between group). Significant at p<0.05. SD: standard deviation.

Group	KleanCREATINE^™^ (n=26)	Marketed CrM (n=26)	P Value	U value
Day 1	8.00±0.69	8.00±0.69	0.992	338
Week 4	7.12±0.59 (11.06%)	7.12±0.65 (11.06%)	0.984	336.5
Week 8	5.88±1.03 (26.4%)	6.92±0.27 (13.46%)	0.007	189

Assessment of Vital Signs and Compliance

No clinically significant changes were observed in blood pressure, pulse rate, respiratory rate, and oral temperature across both the groups throughout the study. All vital signs remained within normal limits in all participants. All participants were 100% compliant to the dosing regimen.

## Discussion

*Phase I*: Pharmacokinetic data showed a higher absorption rate and extent for KleanCREATINE™ versus marketed CrM, indicated by higher C_max_ and mean plasma AUC. KleanCREATINE™ had a shorter T_max_ (two hours) compared to marketed CrM (three hours). Relative bioavailability was significantly enhanced for KleanCREATINE™ (160.42% in sequence 1, 175.45% in sequence 2). Overall, KleanCREATINE™ demonstrated superiority to the marketed CrM.

*Phase II*: A randomized, controlled study compared KleanCREATINE™ to marketed CrM in enhancing strength, power, muscle volumization, energy, and lean body mass in active males over eight weeks. Twenty-six participants (average age 27.37 years) per group followed a mixed (cardio and strength) training protocol four to five times weekly. After eight weeks, body weight and BMI remained unchanged in both groups. KleanCREATINE™ significantly decreased body fat percentage and increased skeletal muscle mass, while marketed CrM showed non-significant changes. These findings closely align with results from a recent meta-analysis, which found creatine supplementation increased lean body mass and reduced body fat percentage during resistance training in adults under 50. Similarly, systematic reviews have shown that site-specific hypertrophy is modestly enhanced by creatine in combination with resistance training [8,9]. Arm circumference increased significantly in both groups, with KleanCREATINE™ showing a higher increase (12.65% vs 6.65%). Thigh circumference increased by 10.36% with KleanCREATINE™ and 4.65% with marketed CrM, without significant difference between the two groups. 

Muscle strength (1RM leg press and chest press) increased significantly in both groups. Lower body strength (leg press) improved from week four, with no significant between group difference after eight weeks. Upper body strength (chest press) improved significantly, with KleanCREATINE™ showing greater improvement at week eight to the marketed CrM. Leg press 50% 1RM endurance significantly improved from week four in both groups. Chest press 50% 1RM endurance significantly increased in both groups, favouring KleanCREATINE™ from day one through week eight. Leg press, chest press, and total repetitions to fatigue at 50% 1RM for leg press and chest press increased significantly by week eight in both groups, with the KleanCREATINE™ showing greater improvements. Research confirms these results, with creatine supplementation enhancing both upper and lower body strength in adults, especially younger individuals [10]. Perceived exertion after workouts decreased from week four and further by week eight in both groups, with greater reductions in the KleanCREATINE™ group.

No clinically significant changes in vital signs were observed, and no adverse events occurred throughout the study, indicating the safety of both products. Additionally, all participants were 100% compliant with the investigational products. In summary, KleanCREATINE™ demonstrated superior efficacy in improving body composition, muscle strength, endurance, and perceived exertion compared to marketed CrM, with both products exhibiting excellent safety profiles.

Creatine is a chemical (amino acid) naturally produced in the body that provides muscles with the energy they need to function correctly. Creatine may also be found in foods such as red meat and seafood. Additionally, many consume creatine supplements to improve their exercise performance and muscle mass. In the liver, some creatine breaks down into creatinine. The kidneys then filter the creatinine out of the blood and expel it from the body in urine. However, some individuals may produce too little or too much creatine due to an underlying health condition or overconsumption of supplements.

Creatine supplementation using the saturation phase followed by maintenance phase is effective for the improvement of performance related to strength, muscular endurance and anaerobic power. However, conflicting data with regard to the effect of creatine supplementation without a “loading phase” and using low doses exist [11]. 

The results showed that creatine monohydrate supplementation increased %t skeletal mass and reduced % body fat in both groups. Previous placebo-controlled studies showed increased lean tissue mass. Another placebo-controlled clinical study revealed that mean percent body fat was not affected by creatine supplementation while there was increased body weight after four weeks of treatment. In contrast, the present study observed reduced body fat and no body weight changes after eight weeks, with significantly higher changes in the KleanCREATINE™ group. Creatine supplementation increases muscle strength and endurance by enhancing phosphocreatine (PCr) resynthesis, which rapidly converts adenosine diphosphate (ADP) to adenosine triphosphate (ATP) during resistance training. This process maintains ATP levels crucial for muscle contractions. In aging adults, exogenous creatine can improve high-energy phosphate metabolism and increase intramuscular PCr, enhancing muscle performance. Additionally, creatine may enhance calcium reuptake in the sarcoplasmic reticulum, leading to faster actin-myosin cross-bridge detachment and potentially increasing muscle force-generating capacity. These mechanisms are well-described in the literature, with studies indicating increased intramuscular PCr and improved ATP availability as driving factors for enhanced performance [12-14].

Oral creatine supplementation increases intramuscular PCr content by 10%-40%, enhancing the ATP-PCr energy system crucial for high-intensity activities up to 30 seconds. Improved PCr availability and resynthesis maintain higher performance during anaerobic activity. Creatine supplementation boosts muscle strength and performance through enhanced high-energy phosphate metabolism, faster actin-myosin cross-bridge cycling, and increased PCr availability, helping maintain ATP levels during intense activities [15].

The present study findings align with previous research showing that post-exercise creatine supplementation increased lean tissue mass more than placebo, and creatine supplementation during resistance training enhanced upper and lower body strength compared to resistance training alone (placebo) [16]. Research studies emphasize the importance of adequate recovery post-workout and its relation to exertion and fatigue. The greater reductions in perceived exertion in the KleanCREATINE™ group might be explained by modulation in muscle activation and afferent feedback in metabolic recovery, such as ATP-CP recharge and lactate removal, which mitigate fatigue [17].

The better pharmacokinetic profile of KleanCREATINE™ compared to the marketed product could support the findings of the present study. Enhanced pharmacokinetic comparisons and formulations with greater peak plasma concentrations and improved bioavailability have been documented for newer creatine supplements [18]. The investigational product is designed for convenient consumption, with greater solubility (being 100% water-soluble) and no residual content. This facilitates faster absorption into the blood, potentially promoting more efficient transport of creatine to the muscles. Overall, KleanCREATINE™ demonstrated superior efficacy in improving body composition, muscle strength, endurance, and perceived exertion compared to marketed creatine monohydrate, with both products showing excellent safety profiles. These findings suggest that KleanCREATINE™ offers slightly higher benefits than the marketed product.

Overall, this study provides evidence that a micronized creatine monohydrate formulation can deliver superior pharmacokinetics and enhanced training-related outcomes compared with a standard creatine product, supporting prior work on enhanced formulations [18,19]. The strengths of the trial include its two-phase design, combining pharmacokinetic and efficacy data, and the use of randomization and blinding in Phase II. Nonetheless, certain limitations must be acknowledged: the modest sample size, restriction to male participants, short intervention duration. Biochemical markers such as intramuscular creatine, lactate, or creatine kinase were not assessed, and including these measures could have provided additional mechanistic insight into muscle metabolism and recovery. These factors may limit the generalizability of the findings. Future studies with larger, more diverse populations, longer follow-up, and incorporation of mechanistic endpoints, such as intramuscular creatine content or biochemical indices related to energy metabolism and recovery are warranted to further validate and extend these results.

## Conclusions

KleanCREATINE™ demonstrated greater bioavailability, faster absorption, and greater efficacy in improving body composition, muscle strength, endurance, and perceived exertion compared with a marketed creatine monohydrate. Both formulations were safe and well tolerated. These findings suggest that micronized creatine monohydrate may offer a more effective supplementation strategy for athletes and physically active individuals. Additionally, the vegetarian-friendly composition, micronized powder form, and United States Pharmacopeia (USP)-grade quality of KleanCREATINE™ make it a versatile and appealing option for athletes and fitness enthusiasts seeking a high-quality creatine supplement.
